# Simple viral/minimal *piggyBac* hybrid vectors for stable production of self-inactivating gamma-retroviruses

**DOI:** 10.1186/s13104-015-1354-y

**Published:** 2015-08-27

**Authors:** Boris Troyanovsky, Vira Bitko, Brian Fouty, Victor Solodushko

**Affiliations:** Exscien Corporation, Mobile, AL 36688 USA; NanoBio Corporation, Ann Arbor, MI 48105 USA; Center for Lung Biology, University of South Alabama School of Medicine, Mobile, AL 36688 USA; Department of Pharmacology, University of South Alabama School of Medicine, Mobile, AL 36688 USA; Internal Medicine, University of South Alabama School of Medicine, Mobile, AL 36688 USA

**Keywords:** Minimal *piggyBac* vector, Self-inactivating retroviral vector, Stable gamma-retroviral production

## Abstract

**Background:**

Transient production of gamma-retroviruses, including self-inactivating (SIN) retroviruses, is a common method for rapidly generating virus capable of gene delivery. *Stable (continuous)* production of virus is preferable to transient production for clinical and biotechnology purposes, however, because it allows for significant quantities of a uniform virus to be generated over a prolonged period of time, thus allowing for longitudinal functional studies and quality analysis. Unfortunately, stable production of SIN retroviruses is difficult to achieve.

**Results:**

We describe a novel method to rapidly and cost-effectively create packaging cells capable of continuously producing self-inactivating gamma-retroviruses. We imbedded the SIN proviral construct into a minimal *piggyBac* transposon vector and then integrated the hybrid vector into packaging cells that already stably expressed the viral gag-pro-pol and envelope genes. Cells that stably produced self-inactivating gamma-retroviruses could be identified (and purified) as early as 3 weeks after initial transfection; these cells produced virus for at least 9 weeks without a decline in titer.

**Conclusions:**

This viral-minimal piggyBac hybrid vector allowed for the rapid generation and purification of packaging cells capable of stably producing self-inactivated gamma-retroviruses. This method can be applied to the large-scale production of viruses for use in research, biotechnology, and potentially, clinical trials.

## Background

Gamma-retroviral vectors were first used in gene therapy clinical trials in 1990 [1–4] and remain an important method for stably introducing genes into cells. While viral production can be accomplished more quickly by the *short*-*term* (transient) transfer of plasmids [5, 6], continuous stable production of virus is highly desirable for clinical and biotechnology purposes [7]. Stable production allows for the large-scale generation of a uniform virus, facilitating its full characterization. It also reduces the labor involved in repeatedly transfecting packaging cells to obtain virus [1].

The use of wild type vectors, in which the viral core is integrated into the packaging cell during the initial infection [7], is an easy method for generating stable gamma-retroviral producing cells. Unfortunately, wild type vectors are more prone to insertional oncogenesis and cell immortalization than self-inactivated (SIN) vectors [8–13] reducing enthusiasm for their use. In addition to a better safety profile, SIN vectors also eliminate internal promoter interference which allows for improved transgene expression [14]. This makes them a better choice than wild type vectors for generating stable virus-producing cells. The absence of an enhancer in both long terminal repeats (LTR) of SIN retroviruses significantly attenuates virus production in packaging cell lines after they are integrated, however [15]. Therefore, while creating stable SIN retroviral-producing cells is possible, it is tedious and time-consuming [16].

Recently we developed a modified *piggyBac* transposon delivery system in which the transposase gene and most of both terminal domains were relocated from the delivery cassette into the helper (non-integrating) part of the same plasmid without a significant loss of transposition efficiency [17]. Similar to classical *piggyBac* vectors, these truncated (minimal) transposon vectors can deliver a large amount of transgenic DNA, yet incorporate significantly less helper DNA (fewer than 100 base pairs) than classical *piggyBac* vectors or retroviruses. In addition, this minimal *piggyBac* system has the advantage of requiring only a *single*-plasmid for delivery. This manuscript describes the practical application of this minimal piggyBac vector system for the large-scale manufacture of clinical-grade, self-inactivating gamma-retroviral vectors. We used this minimal *piggyBac* delivery system to efficiently integrate SIN retroviral sequences and genes necessary for cell selection such as antibiotic resistance or fluorescent markers into packaging cells. By incorporating a selection marker within the integrated transposon, but outside the gamma-retroviral sequence, we were able to rapidly select only the cells that had integrated the entire provirus and thus, able to stably produce SIN virus.

## Methods

Phoenix (HEK293 backbone) amphotropic packaging cells were purchased from Orbigen, Inc. (San Diego, CA). They were used for transfection and infection experiments as virus producer and target cells. Cells were cultured in DMEM, 10 % FBS, 2 mmol l-Glutamine and routinely passaged after reaching 80 % confluency. All cells were grown in humidified incubators at 37 °C in 5 % CO_2_ and harvested by 0.05 % trypsin/0.53 mM EDTA digestion and counted with Coulter Z1 (Coulter Electronics). Counts were made in triplicate.

RNAi-Ready pSIREN-RetroQ-ZsGreen Vector was purchased from ClonTech (Mountain View, CA). It is a self-inactivating (SIN) retroviral expression vector due to a truncation in the 3′ LTR enhancer/promoter region (U3). The hybrid 5′ LTR consists of the cytomegalovirus (CMV) type I enhancer and the mouse sarcoma virus (MSV) promoter. A BglII-BamHI deletion of the U6 promoter and ligation of the vectors ends with a linker to recover EcoRI site generated *plasmid B*. We then replaced the truncated U3 fragment of the 3′LTR in plasmid B (XhoI-SacI restriction sites fragment) with a full-length U3 part of the 3′LTR from the retroviral vector pBMN-GFP (BsrGI-SacI fragment) (purchased from Orbigen, San Diego, CA) to generate *plasmid A*. This transformed the SIN 3′LTR in retroviral plasmid B into a wild type 3′LTR, while preserving all other viral sequences including the hybrid CMV/MSV/5′LTR, packaging sequence (Ψ) and the CMV driven ZsGreen identical to those in plasmid B. An SspI-EcoRI deletion in plasmid B generated control *plasmid D*.

**Fig. 1:**
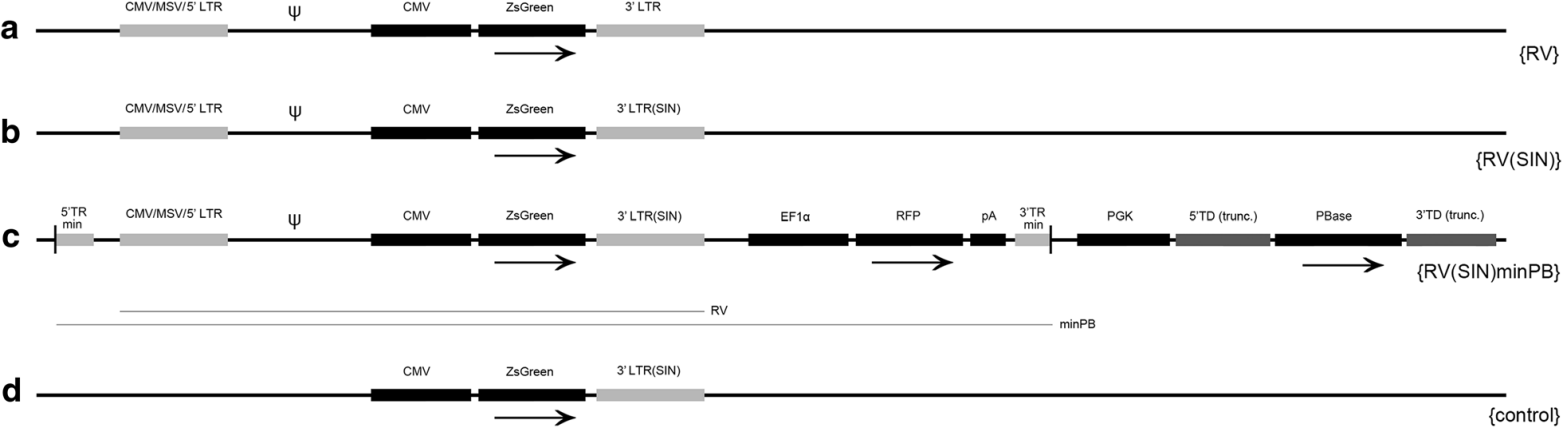
Detailed schematic of plasmids. All four plasmids contained a ZsGreen reporter gene driven by a cytomegalovirus (CMV) promoter. Functional retroviral sequences required for virus production (indicated by the *line* designated RV) are present in plasmids *A*, *B*, and *C*. Control plasmid *D* lacked the 5′ LTR and the packaging sequence (Ψ) and therefore was not able to generate virus, but expressed ZsGreen. In hybrid plasmid C the retroviral sequence was imbedded into the minimal *piggyBac* vector (*line* designated as minPB). The reporter gene, RFP (turboRFP—Red Fluorescent Protein) is driven by the EF1α promoter. *PiggyBac* transposase and truncated *piggyBac* terminal domains are required for minimal *piggyBac* integration, but these sequences do not integrate into target chromatin. Arrows indicate the orientation of the operons. Prokaryotic origin of replication and *ampicillin* resistance gene are not shown. (Vectors are aligned for easier comparison, but distances are not drawn to scale). *LTR* full-length wild type gamma-retroviral long terminal repeat, *CMV/MSV/5′LTR* hybrid 5′ LTR consists of the CMV type I enhancer, *MSV* the mouse sarcoma virus promoter linked to the truncated part of the retroviral long terminal repeat, *3′LTR(SIN)* self-inactivated retroviral long terminal repeat, *TRmin* minimal piggyBac terminal repeat, *pA* SV40 polyadenylation signal, *BPase* *piggyBac* transposase gene driven by the PGK promoter, *TD(trunc.)* truncated piggyBac terminal domain

The vector 166 (described in Ref. [17]) was used as the base for constructing the hybrid minimal *piggyBac*/retroviral *plasmid C*. Vector 166 was modified by replacing the minSV40 promoter for *piggyBac* transposase expression and the CMV promoter driving turboRFP expression for PGK and EF1α promoters respectively (Fig. 1c). The entire SIN retroviral construct from plasmid B (SspI-DraI restriction sites fragment) was inserted into a BamHI site in the modified plasmid 166 in the same orientation as the turboRFP and *piggyBac* transposase genes to make plasmid C.

**Fig. 2:**
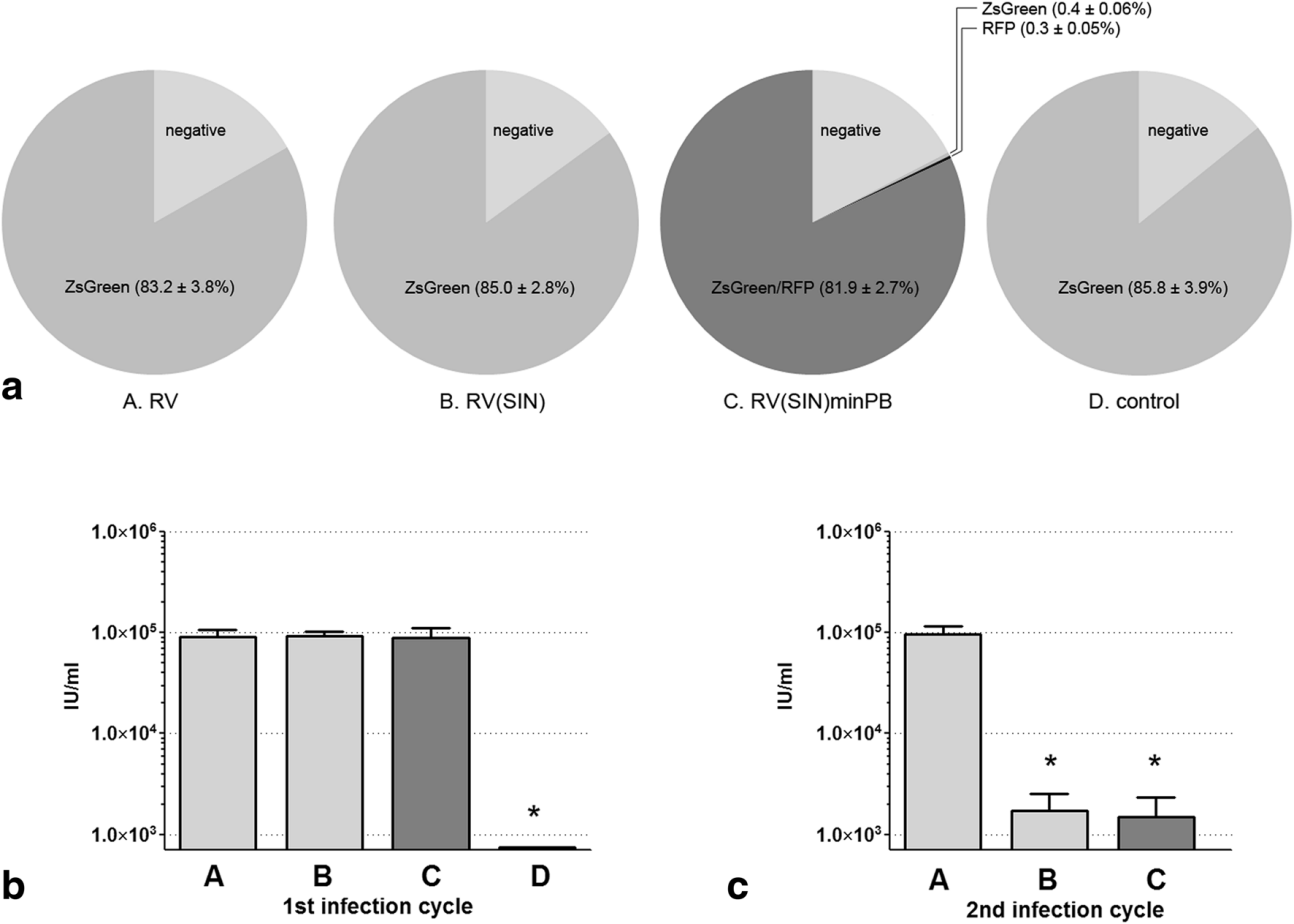
**a** Percentage of *ZsGreen*, turbo*RFP* or *ZsGreen*/turbo*RFP* positive packaging cells 48 h after transfection (i.e. transiently positive) with tested plasmids. *RV* wild type retroviral vector *A*, *RV(SIN)* SIN retroviral vector *B*, *RV(SIN)minPB* SIN retroviral/minimal piggyBac hybrid vector *C*, *Control* negative control vector *D*. **b**, **c** The viral titers (*IU* infectious units/ml) in the medium of two sequential passages of retrovirus originated from transiently transfected plasmids. Viral conditioned medium was collected from virus-producing cells 48 h after transfection with plasmids *A*–*D* and then applied to other packaging cells (1st infection cycle, **b**). Only cells expressing the viral marker (ZsGreen) after the 1st infection cycle were purified and their conditioned medium tested for retroviral SIN function using new target cells (2nd infection cycle, **c**). (Data are expressed as mean ± SE; *n* = 4 experiments; *P < 0.05)

**Fig. 3:**
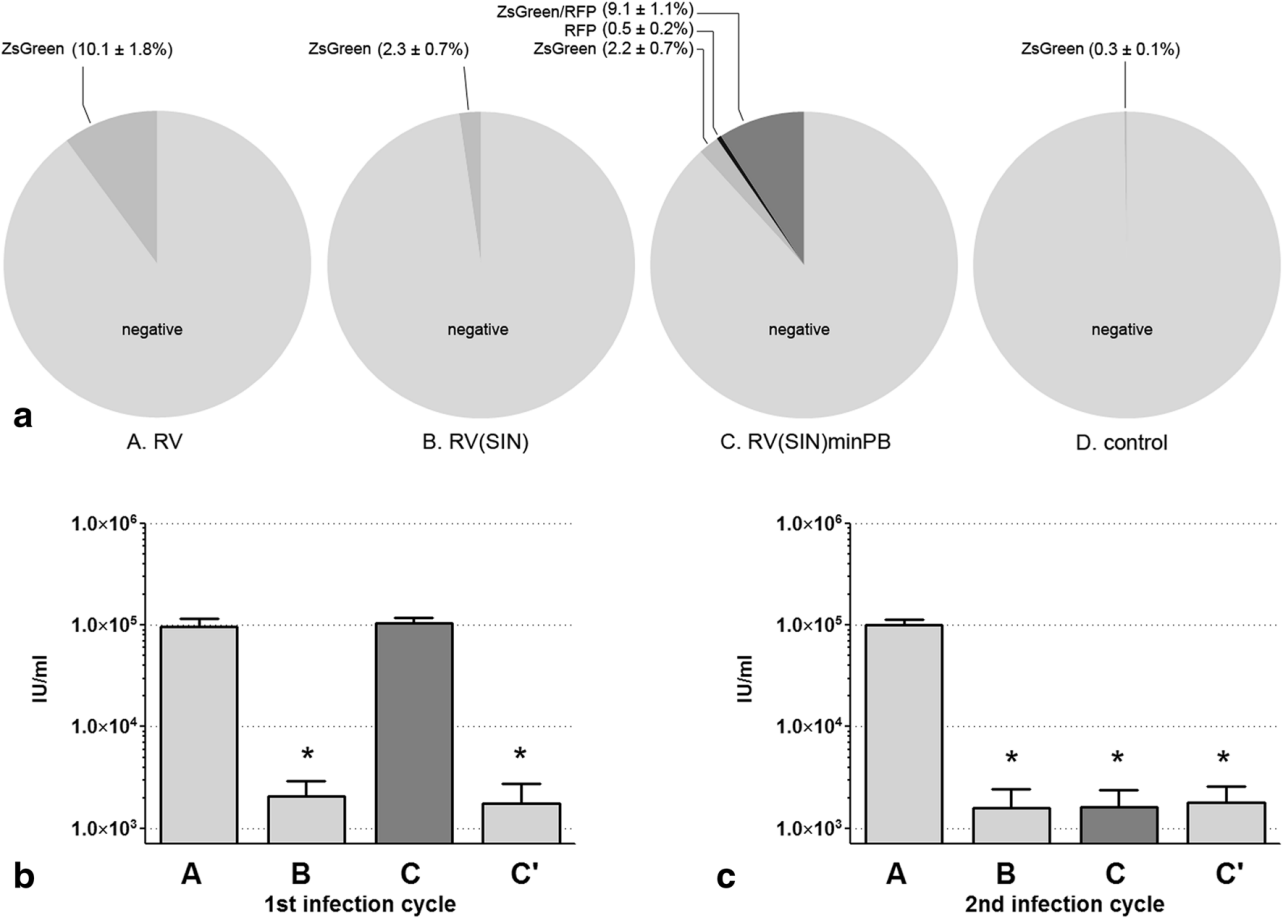
**a** Percentage of *ZsGreen*, turbo*RFP* or *ZsGreen*/turbo*RFP* positive packaging cells 21 days after transfection (i.e. stably positive) with tested plasmids. *RV* wild type retroviral vector *A,* *RV(SIN)* SIN retroviral vector *B,* *RV(SIN)minPB* SIN retroviral/minimal piggyBac hybrid vector *C*, *Control* negative control vector *D*. **b**, **c** The viral titers (*IU* infectious units/ml) in the medium of two sequential passages of retrovirus originated from stably integrated constructs. Twenty-one days after transfection with the three retroviral plasmids, only virus-producing cells expressing ZsGreen (populations *A*, *B* and *C*’) or both ZsGreen and turboRFP (population *C*) were purified and their conditioned medium applied to other packaging cells for infection (1st infection cycle, **b**). Only cells expressing the viral marker (ZsGreen) after the 1st infection cycle were purified and their conditioned medium tested for retroviral SIN function using new target cells (2nd infection cycle, **c**). (Data are expressed as mean ± SE; *n* = 4 experiments; *P < 0.05)

Amphotropic packaging cells were transfected with these test plasmids using FuGENE HD reagent following the manufacturer’s instructions for virus production (each expressing ZsGreen with excitation/emission of 496/506 nm; plasmid C also expressing turboRFP with excitation/emission of 553/574 nm). Forty-eight hours or 21 days after transfection, the cells were harvested by 0.05 % trypsin/0.53 mM EDTA digestion, washed, and analyzed/sorted (Figs. 2a, 3a) using a BD Biosciences FACSAria cell sorter in the University of South Alabama Flow Cytometry Core. The sorted cells were re-seeded for virus production experiments for an additional 9 weeks. Viral-conditioned media was refreshed and then collected 12 h later; it was then filtered, supplemented with hexadimethrine bromide (polybrene) to a final concentration of 4 µg/ml and applied directly to target cells. The cell density of virus-producing and target cells was the same for all reported transfection/infection experiments. To titrate virus, we applied 10 ml of increasingly diluted conditioned medium to 1.5 × 10^6^ target cells and determined infection efficiency using flow cytometry. The viral titer was calculated within the linear range based on the percentage of ZsGreen-positive cells over several virus dilutions. For other experiments, we applied 10 ml of medium to 4 × 10^6^ target cells and calculated viral titer within the linear (i.e. non-saturating) range of viral infectivity.

Data are expressed as mean ± SE. Changes in percentage of ZsGreen, turboRFP or ZsGreen/turboRFP-expressing cells, and virus titer were compared using ANOVA combined with Fisher post hoc analysis, with a *P* value <0.05 considered significant.

## Results and discussion

We used minimal *piggyBac* transposon vectors to efficiently deliver a SIN gamma-retroviral core into the packaging cell genome to allow for stable virus production. To begin, we constructed three gamma-retroviral plasmids each containing the fluorescent marker, ZsGreen, under control of the CMV promoter (Fig. 1): plasmid A was a wild type retrovirus with an intact 3′ LTR; plasmid B was a SIN retrovirus (with a truncated 3′LTR); and plasmid C was a hybrid minimal *piggyBac*/SIN-retroviral vector that included the entire SIN proviral construct (identical to Plasmid B) and also contained turboRFP as a transposon reporter. Plasmid D lacked both the 5′ LTR and the retroviral packaging sequence and thus was unable to generate virus; it was used as a negative control. Cell density and medium volumes in all experiments were kept similar to allow us to calculate variations in viral titer for each of the vectors.

The initial transfection efficiency of all three retroviral (A–C) and control (D) plasmids in Phoenix amphotropic packaging cells (Orbigen, San Diego, CA), as determined by the percentage of ZsGreen positive cells (or ZsGreen/turboRFP positive cells for plasmid C) 48 h after transfection, was similar (approximately 84 %; Fig. 2a); the fact that plasmid C was 2kB larger than the other plasmids had no effect on transfection efficiency. Conditioned medium was collected from these transfected cells at 3 days and then applied to other amphotropic packaging cells to determine their viral titer (1st infection cycle). Conditioned medium from cells that had integrated virus after the 1st infection cycle (as determined by ZsGreen positivity after infection) was collected and applied to other amphotropic packaging cells to determine their viral titer during a 2nd infection cycle. This experimental design allowed us to determine if virus infectivity could be maintained through both infection cycles.

Conditioned medium harvested 3 days after transfection with the three retroviral plasmids (A–C) demonstrated an equivalent viral titer (Fig. 2b); cells transfected with plasmid D did not make virus and therefore were not analyzed further. As expected, only conditioned medium collected from cells infected with the wild type virus (virus A) was able to infect cells during a 2nd infection cycle with the same efficiency as that of the parental cells (Fig. 2c, A). We did observe a low-level production of retrovirus in cells infected with the SIN virus in the 2nd infection cycle, however (Fig. 2c, B, C). This was most likely due to reactivation of the 5′ LTR(SIN) promoter in some cells as previously suggested by other investigators [18].

Our goal was to create packaging cells capable of *stably* generating SIN gamma retroviruses, however. This required the integration of the SIN provirus into the packaging cell genome without losing its ability to produce infectious viral particles. To determine whether an integrated SIN provirus could stably produce virus, we examined viral titers of conditioned media 3 weeks after the initial transfection. We chose this time-point because we have previously demonstrated [17] that HEK293 cells either integrated or eliminated the minimal *piggyBac* constructs within 3 weeks of transfection. Therefore, transfected cells that continued to express ZsGreen at 21 days were assumed to have incorporated the viral construct. 10.1 % of cells transfected with plasmid A (wild type) and 2.3 % transfected with plasmid B (SIN) were ZsGreen positive at 21 days. The higher percentage of ZsGreen cells in wild type-infected cells was likely due to the continued infection of cells by the integrated wild type provirus which was capable of making active virus. In contrast, the integrated SIN provirus was not capable of generating infectious particles, so there was minimal cross infection in culture leading to fewer ZsGreen-positive cells. Cells transfected with plasmid C generated two different populations of cells at 21 days: population C’ (*ZsGreen*-*only positive* indicating cells that had integrated only the retrovirus during the 21 days, likely through cross infection) and population C (*both ZsGreen and turboRFP positive* indicating cells in which the *entire* transposon, with the imbedded retrovirus, had been integrated into the cell). Cells expressing these fluorescent markers 21 days after transfection (Fig. 3a) were purified by flow cytometry and studied further. This gave a total of four sub-populations of cells (A, B, C, and C’).

**Fig. 4:**
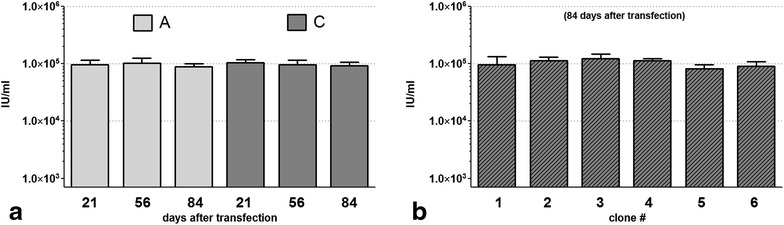
**a** Viral titer (*IU* infectious units/ml) in conditioned medium from infected, but non-clonal populations of cultured packaging cells over time. Viral-producing cells that contained either the stably incorporated wild type retroviral construct (expressing ZsGreen, population *A*) or the hybrid-SIN vector (expressing both ZsGreen and turboRFP, population *C*) were obtained 21 days after transfection and cultured for an additional 35 and 63 days (56 and 84 days after transfection). Conditioned media was collected at the indicated time points and the viral titer compared to the titer at 21 days. *A* cells that integrated wild type retrovirus by infection; *C* cells that integrated the SIN retrovirus-piggyBac hybrid by transposition. **b** Viral titer (*IU* infectious units/ml) in conditioned medium from six randomly generated clones of packaging cells 84 days after transfection with plasmid *C*. Viral-producing cells that stably incorporated the minimal piggyBac/SIN retroviral construct (as determined by cells that expressed both ZsGreen and turboRFP (population *C*) 21 days after transfection) were purified and propagated in culture for an additional 63 days. The viral titer in the conditioned media from each clone at this time was determined as previously described for the non-clonal population of cells. (Data are expressed as mean ± SE; *n* = 4 experiments)

These four populations of cells were used for two cycles of virus propagation/collection/infection/analysis experiments as described previously in this paper. Only cells that had integrated the wild type virus (A) or those that had integrated the hybrid minimal piggyBac/SIN gamma-retroviral construct (C) were able to produce sufficient levels of virus in this 1st round of infection (Fig. 3b). As expected, only viral vector A was able to produce virus capable of infecting target cells in the 2nd amplification/collection/infection cycle (Fig. 3c). The inability of double-positive cells (C) to produce virus that remained infectious beyond a single passage indicated that the SIN function of the integrated retrovirus was retained. Both the wild type (A) and the SIN (C) retroviral producing cells were able to maintain virus production, without any appreciable drop in titer, for at least 63 days after purification by flow cytometry (Fig. 4a), sufficient time to not only fully characterize the virus, but also to produce it on a scale suitable for most biotechnology and clinical applications. Table 1 summarizes the use of the tested vectors in transient and stable virus production formats.

**Table 1:** Wild type (WT) and self-inactivated (SIN) virus production by the used vectors

Vector	Production format	
	Transient	Stable
A: RV	WT	WT
B: RV(SIN)	SIN	–
C: RV(SIN)minPB	SIN	SIN
D: Control	–	–

We used separate reporter markers for the viral and the non-viral parts of the transposon (hybrid) vector. This significantly improved our ability to distinguish between cells that had incorporated the entire hybrid minimal piggyBac/SIN gamma-retroviral construct (and were thus capable of generating infectious SIN gamma-retrovirus) from those that had only integrated the retrovirus (and thus unable to generate infectious SIN gamma retrovirus). Integration of transposons, like integration of retroviruses, is a random event, however. Therefore, even within the population of cells that had integrated the entire piggyBac/SIN gamma-retroviral construct, the site of integration was not likely to be the same in each cell. Over time this could lead to variable virus production if certain cells overgrew the population. To determine whether this occurred in these cells, we randomly generated six individual clones that expressed both fluorescent markers (ZsGreen and TurboRFP) 21 days after transfection and expanded them in culture. Eighty-four days after transfection, all of the six clones produced virus with a similar titer to that from a non-clonal cell population also cultured for total of 84 days (after transfection) (Fig. 4b). This result suggests that in mixed (non-clonal) infected populations of packaging cells, the majority of cells make virus at comparable rates; thus, cloning of infected cells is not required, reducing the cost and time of generating these packaging cells.

In these experiments we used the same cells for both virus production and targeting which allowed us to compare the first (transient) and second (stable) cycles of virus infectivity. However, in packaging cells that already stably express viral GAG-POL and envelope proteins, re-infection occurs with a lower efficiency [7]. This likely explains the lower viral titer in these cells compared to HEK293 cells without these viral proteins. Yet, despite this inhibition, the viral titers obtained are comparable to those used in the manufacturing of clinical-grade SIN gamma-retroviral vectors [6, 19]. The infectivity of gamma-retroviruses depends on the susceptibility of specific target cells to infection and also can be affected by other enhancing technologies [20, 21]. The methods described in this manuscript technique are relevant only to gamma-retroviral vectors since the continuous expression of the lentiviral GAG-PRO is known to be toxic to packaging cells [22].

Minimal *piggyBac* vectors [17] can incorporate DNA sequences up to (at least) 15 Kb into cells (unpublished data) allowing the stable delivery of large retroviral sequences. Using this vector to deliver the SIN retroviral construct increased the efficiency of proviral DNA incorporation into virus producing cells more than 30-times compared with the use of non-transposon stable plasmid delivery systems [from 0.3 ± 0.1 (ZsGreen positive cells, plasmid D) to 9.1 ± 1.1 % (ZsGreen/RFP double positive cells, plasmid C); Fig. 3a]. Usual piggyBac vectors can also be used for gamma-retroviral delivery [23]. Two important advantages of using a minimal rather than a classical *piggyBac* vector, however, are that the minimal vector is a *single* plasmid system which allows for more efficient delivery and that transposons integrated into cells remain non-removable even in the presence of *transposase.* Thus, minimal piggyBac vector can be used repeatedly for multiple integration events without the risk of losing previously integrated fragments. No additional components, such as helper plasmids, proteins or mRNAs (commonly used with traditional transposon systems) are needed. The proposed system provides a continuously-producing, highly-consistent cell system that can be used to generate SIN retroviral vectors that meet the standard for current Good Manufacturing Practices [1, 2, 24].
